# Virulence during Newcastle Disease Viruses Cross Species Adaptation

**DOI:** 10.3390/v13010110

**Published:** 2021-01-15

**Authors:** Claudio L. Afonso

**Affiliations:** Base2bio, LLC, Oshkosh, WI 54905, USA; claudio.afonso@base2bio.com; Tel.: +1-800-817-7160

**Keywords:** NDV, evolution, virulence, host adaptation

## Abstract

The hypothesis that host adaptation in virulent Newcastle disease viruses (NDV) has been accompanied by virulence modulation is reviewed here. Historical records, experimental data, and phylogenetic analyses from available GenBank sequences suggest that currently circulating NDVs emerged in the 1920–1940′s from low virulence viruses by mutation at the fusion protein cleavage site. These viruses later gave rise to multiple virulent genotypes by modulating virulence in opposite directions. Phylogenetic and pathotyping studies demonstrate that older virulent NDVs further evolved into chicken-adapted genotypes by increasing virulence (velogenic-viscerotropic pathotypes with intracerebral pathogenicity indexes [ICPIs] of 1.6 to 2), or into cormorant-adapted NDVs by moderating virulence (velogenic–neurotropic pathotypes with ICPIs of 1.4 to 1.6), or into pigeon-adapted viruses by further attenuating virulence (mesogenic pathotypes with ICPIs of 0.9 to 1.4). Pathogenesis and transmission experiments on adult chickens demonstrate that chicken-adapted velogenic-viscerotropic viruses are more capable of causing disease than older velogenic-neurotropic viruses. Currently circulating velogenic–viscerotropic viruses are also more capable of replicating and of being transmitted in naïve chickens than viruses from cormorants and pigeons. These evolutionary virulence changes are consistent with theories that predict that virulence may evolve in many directions in order to achieve maximum fitness, as determined by genetic and ecologic constraints.

## 1. Introduction

Newcastle disease viruses (NDVs), also known as avian paramyxoviruses type 1, are members of the genus *Orthoavulavirus*, species Avian *orthoavulavirus* 1 in the family Paramyxoviridae. They are negative-sense, single-stranded, non-fragmented RNA viruses, distributed worldwide, and they are able to infect wild, peridomestic and domestic avian species. NDVs are highly adaptable viruses, capable of infecting over 200 avian species and of causing a diverse range of clinical outcomes in birds [1]. The NDV genome is composed of a single-stranded RNA molecule; it encodes for at least six proteins arranged in a 3′ to 5′ order: nucleocapsid protein (NP), phosphoprotein (P), matrix protein (M), fusion protein (F), hemagglutinin-neuraminidase (HN), and RNA polymerase [2,3].

Two accessory proteins (V and W) with a possible role in virulence have been described for NDV. These proteins are generated by RNA editing of the genomic region corresponding to the phosphoprotein gene region. Although the role of the W protein is still controversial, the role of the V protein in modulating virulence through a process that involves targeting the phospho-STAT1 degradation to block IFN-α signaling has been demonstrated [4,5,6,7,8]. In a more recent manuscript, a comprehensive description of the genetic variations and the molecular evolution of the P gene-edited and the accessory viral proteins is presented [9]. A role of the V protein in host range restriction is expected as it has been shown in vitro studies with human cells that it may play a role affecting host range. However, a study on the role of the V protein on evolution during the process of natural host adaptation or a study of the role of the different V protein variants on fitness to replicate in different hosts remains to be done and will not be reviewed here.

Although every protein of the virus is likely to interact with the host in one manner or another, none have been studied more deeply than the fusion protein [10,11,12,13,14,15]. This protein allows the virus membrane to fuse to the host cell membranes during infection. The amino acids at the fusion protein cleavage site interact with secreted host mucosal proteases, which cleave the fusion protein precursor into an active form, making it possible for the virions to attach, infect and replicate in multiple tissues and organs. This enhanced capacity of the cleaved fusion protein is responsible for increased virulence [11,16,17]. Viruses that lack phenylalanine and one or more of the necessary basic amino acids at the cleavage site are normally not able to systemically spread, and they are limited to replicating in the upper respiratory and enteric tracts, thus being considered to be viruses of low virulence, also described as lentogeneic or lNDV.

There are a large variety of pathogenicity tests to evaluate NDV virulence, and these are used to determine if NDVs are capable of producing disease in naïve chickens. These tests range from evaluation of the pathogenicity in eggs (mean death time, MDT), or in one-day-old naïve chicks (intracerebral pathogenicity index, ICPI), to an evaluation in adults (vaccinated and naïve challenge models on mature SPF birds) [2,16,18,19]. Lentogens or lNDV have ICPI ≤ of 0.7, and they generally produce no clinical signs or subclinical infections with minimal respiratory involvement, whereas those of moderate pathogenicities, such as mesogens or mNDV with ICPI values of 0.7 to 1.5, generally cause clinical signs of disease, but typically result in non-lethal outcomes in chickens. Viruses of high pathogenicity, called velogens o vNDV, have ICPI > 1.5 and generally cause serious disease and mortality among naïve chickens. There are two types of velogens (neurotropic or vnNDV and viscerotropic or vvNDV) that cannot be clearly distinguished with the ICPI tests and require intravenous or clinical disease tests on adult birds for differentiation [2,20]. For vaccinated chickens, infection with viruses of lNDV and mNDV pathotypes is asymptomatic, and infection with vNDV may occasionally cause disease with low mortality if the levels of immunity obtained during vaccination are not satisfactory. Vaccination reduces but does not eliminate viral replication of vNDV.

NDVs display large genetic diversity, which determines the existence of two distinct classes of viruses (class I and II) [21,22]. While all NDVs are capable of infecting chickens, only class II virulent viruses are normally capable of causing disease. Class I viruses are less diverse with only one genotype, while class II viruses are the most diverse, of remarkable economic impact, and will be further analyzed here from the point of view of host adaptation. Lentogenic viruses of class I and II are relatively common in waterfowl, gulls, and shorebirds and generally do not cause clinical disease in chickens [23,24,25,26,27]. Among class II viruses, virulent viruses are the most studied because they cause significant mortality in naïve animals, drop in egg production, and are repeatedly isolated in vaccinated poultry in endemic regions of the world [3,20]. For the most virulent viruses, immune responses attributed to vaccination are not sufficient to prevent mucosal viral replication, and viral shedding occurs at reduced levels [28,29,30]. Therefore, virulent viruses are still capable of disseminating in domestic birds worldwide, occasionally spilling over into wild birds. Virulent viruses of intermediate virulence are often maintained in wild birds such as those which are frequently isolated from double-crested cormorants (genotype XIX) in North America and Canada and occasionally from pelicans and gulls that are in close proximity to cormorants during outbreaks [31,32,33,34]. Other class II viruses with mesogenic and velogenic neurotropic phenotypes of genotypes VI and XXI are preferentially isolated from wild and domestic Columbiformes [35,36,37,38,39,40] and occasionally from spillover into poultry or other domestic birds. Velogenic viscerotropic (vvNDV) of class II genotypes V, VII, XII, XIII, XIV, XVII and XVIII are of more recent origin, are distributed worldwide and are most frequently isolated in poultry [21]. Neurotropic viruses (vnNDV) were most commonly isolated in poultry before the 70s and less frequently since then [41,42,43]; however, they have been maintained in cormorants in the wild. Except for old viruses of genotype V in psittacine, there have not been repeated isolations of vvNDV from wild birds; thus, the existence of natural wild bird reservoirs for vvNDV remains to be demonstrated.

## 2. Review of Supporting Data

### 2.1. NDVs Uniqueness to Study Virulence as a Host Adaptation Mechanism

The hypothesis that the large capacity of NDVs to infect and replicate among multiple avian species is accompanied by a capacity to develop genomic changes that modulate and fine tune virulence in order to achieve host adaptation, is analyzed here. NDVs are unique examples in the study of the relationship between virulence and host adaptation for the following reasons: (1) Most NDVs are known to be able to infect more than one different avian host species; (2) NDVs’ genetic changes associated with virulence have been identified; (3) NDVs’ lineages associated with specific hosts have been identified, (e.g., waterfowl class I, cormorants, pigeons and chickens viruses tend to develop into host specific lineages); (4) There are very good tests to determine virulence; (5) The virulence of a large number of viruses of different origin has already been characterized; (6) There are existing databases of genomic sequences accompanied by clinical data from the 1930s through present day.

Low virulent forms of NDV likely existed and evolved over centuries in wild birds; however, science was only able to document evidence of the presence of NDV when large flocks of chickens started to be raised for their meat in the 1920s. There is evidence that poultry domestication may have started over 3000 years ago when Red Jungle Fowl, a bird whose native territory stretches from east India to Malaysia, dispersed to India China, Egypt, Greece, and Rome [44]. However, the first documented case of disease caused by NDV dates back to the 1920s. Thus, some caveats of this analysis are that the historical record is incomplete as most existing samples are from the recent origin and that most viruses were obtained from opportunistic sampling rather than from planned, systematic surveillance. Another caveat of this study is that there are exceptions to the observed host-pathotypes associations as the virus has a capacity to spill over into non-host species. Thus, with limitations, NDVs still provide 94 years of records, well-characterized genotype-phenotype associations and a fairly well tracked phylogenetic classification, with isolation dates and information on the hosts of origin, to study the evolution of virulence during host adaptation.

### 2.2. Types of Genetic Changes Seen during NDV Evolution

As with other RNA viruses, NDVs are expected to adapt to evolutionary constraints by developing genomic changes that facilitate their replication and maintenance [45,46]. With limitations in the maximum genomic size by the nucleic acid packing capacity of virions and limitations on the capacity to recombine that are normally observed in most negative-stranded genomes, genomic changes in NDVs are normally single nucleotide polymorphism and occasionally deletions/insertions of six or a multiple of six nucleotides [47,48]. Three types of changes have been best characterized in NDVs: (1) changes in the region encoding the fusion protein cleavage site; (2) progressive point mutations at multiple genome sites that lead to amino acid changes on the virion proteins and may confer escape from the immune system or increased fitness for replication and transmission; (3) Codon usage adaptation changes. The combination of changes at the cleavage site (Type 1) and overall genomic adaptations (Types 2 and 3) are most likely to determine host adaptation [49].

Changes in virulence at the cleavage site are rapid, punctual changes in a genomic region that is no longer than 15 nucleotides but is capable of causing drastic changes in virulence [49]. Virulent NDVs normally have three or more basic amino acids (arginine or lysine) at positions 113 to 116 and phenylalanine at position 117; whereas, strains predicted to be of low virulence have fewer than two basic amino acid residues at those positions, with leucine at position 117 [11,50,51]. Changes at the fusion cleavage site causing phenotypic changes from lentogenic to virulent have historically been an issue of concern for poultry. However, without diminishing its importance, the rapid rate of evolution of existing virulent class II NDV genotypes into new genotypes and their increased pathogenicity are presently of more concern than changes at the cleavage site, especially since it has been shown that amino acids at the fusion protein cleavage site suffer strong, negative, selective pressure to remain unchanged [49].

The hypothesis that lentogenic viruses from wild birds were the ancestral origin of some older virulent viruses is fairly well supported by evidence. The origin of older virulent NDVs genotypes could be explained in two different ways: (1) multiple separate changes in the fusion virulence cleavage sites occurred on different existing lentogenic genotypes circulating during the 1920s to the 1940s, (2) a change in virulence at the fusion site in a single unknown lineage, occurring further back in time was followed by genetic diversification of this lineage into other old and new virulent genotypes.

The first possibility is supported by the presence of lentogenic viruses that are very closely related to virulent viruses. For class II genotype II viruses circulating in the United States, the lentogenic LaSota and Hitchner/B1 in 1946 and 1947, respectively, present only minor nucleotide differences when compared with old contemporary virulent viruses of class II genotype II such as Texas/GB/1948, Beaudette C/1945, and others virulent viruses that were collected in the 1940s and the 1950s in the United States [42]. These minor differences suggest that a fusion cleavage site switch responsible for increased virulence occurred shortly before the isolation of virulent viruses in the 1940s. This possibility is further supported by the existence of genotype X viruses in wild birds [22]. This genotype, which phylogenetically shares a common ancestor with virulent viruses of genotype II, is genotype II’s most closely related low virulence lineage and is still circulating in wild birds. Thus, one could predict that viruses of genotype X may still have some potential to become virulent under proper evolutionary pressures.

Another well-documented piece of evidence of the change in virulence at the cleavage site is represented by the outbreak of NDVs of genotype I in Australia during 1998–2001 [52,53]. Researchers there identified in poultry farms lentogenic viruses of genotype I present in mixed infections with nearly identical virulent viruses. A few nucleotide changes at the cleavage site were found in different isolates obtained from chickens’ farms, and thus, they could trace the change in virulence to few cumulative changes at the region encoding the fusion protein cleavage site.

As the origin of other older virulent lineages (III, IV, IX, and XI) is not as well documented, the second hypothesis is only indirectly supported by the evidence that other more recent virulent viruses appear to have originated by evolution from older virulent viruses [22]. The existence of ancestral lentogenic viruses closely related to viruses from those genotypes has not been shown, however the lack of sampling before the 1940s may justify this deficiency. The second hypothesis implies that some of these old genotypes (III, IV, IX, and XI) arose from one single virulent virus. This possibility would be consistent with the evolutionary pattern of modern viruses that have shown rapid evolution from one virulent genotype into another. However, the approximately 10% difference at the nucleotide level among these older genotypes would suggest that either the original change in virulence occurred decades before the representative viruses were isolated, or that accelerated evolution has occurred from an ancestral virulent virus in those genotypes. It is possible that the rate of evolution of viruses under primitive poultry rearing methods (with no biosecurity, multiple points of contact with wild birds, and no vaccination) may have been different from current evolutionary rates, explaining a more contemporary origin. However, to better support this theory additional data in the form of nucleotide sequences from old viral isolates is needed. Both are plausible hypotheses that need to be confirmed by finding nucleic acids from samples preserved from early in the XX or late in the XIX centuries, most likely only available in fixed museum samples.

**Figure 1:**
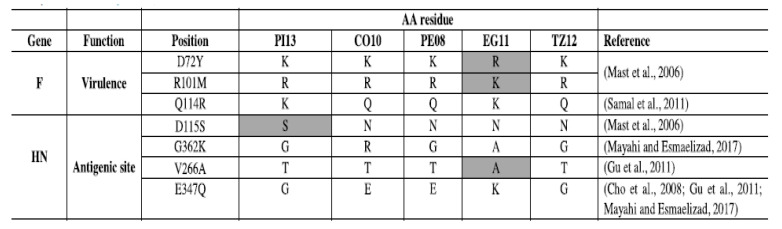
Amino acid changes in viruses of different genotypes. Excerpts from Ferreira, H.L.; Taylor, T.L.; Dimitrov, K.M.; Sabra, M.; Afonso, C.L.; Suarez, D.L. Virulent Newcastle disease viruses from chicken origin are more pathogenic and transmissible to chickens than viruses normally maintained in wild birds [55].

The second type of genomic changes are progressive, cumulative nucleotide changes leading to different types of host fitness adaptations at the protein level, including neutralization escapes, among others [54]. Genotype-associated amino acids mutations and neutralization escape mutants have been identified on many occasions [55,56,57,58] supporting the existence of adaptations at the protein level across the genome. Genotype-specific genetic changes in pigeons and cormorant viruses are reflected by amino acid changes associated with key residues at the fusion and hemagglutinin surface proteins have been shown and are illustrated in Figure 1 [55]. The authors identified genotype associated amino acid changes in sites involved in virulence or antigenicity when comparing an Egyptian virus (EG11) from genotype VII, a Peruvian virus from genotype XII (PE08), a Tanzanian virus (TZ12) of genotype XIII, a cormorant virus (CO10) classified as XIX, and a pigeon adapted virus (PI13) that belongs to genotype XIX. However, despite the identification of these specific genetic changes, the role of individual mutations on host range remains largely unstudied.

The third type of genomic changes are codon usage adaptations defined as the selective use of codons by the viral genome to better adapt to the availability of amino acids produced by the host. It is known that codon usage is not identical among animal species. Thus, during the process of host adaptation, viruses may improve their fitness to replicate in a particular host by changing the use of codons necessary to translate proteins in order to utilize efficiently the host transfer RNAs [59]. Nonrandom codon usage is common in nature, and viral adaptation to available nucleotide pools is likely to be used to regulate protein expression negatively or positively [60,61]. As NDVs are reliant on the avian host’s translational machinery to synthesize the necessary proteins, it would make sense for viruses that are normally isolated from different types of host species to adapt in order to use host’s tRNAs effectively. As NDVs adapts to be maintained from wild bird hosts to chickens, it would be expected that these adaptations would include codon usage adaptations.

A study by Taylor and others [62] included the coding sequences of 175 of the class I fusion genes containing 96,775 codons, 1166 sequences of the fusion gene of class II viruses containing 644,798 codons, the coding sequences of 29 complete genomes of class I containing 132,675 codons, and 259 complete genomic sequences of class II containing 1,184,925 codons. When the codon usage of viruses of low virulence of class I, which are known to be maintained in waterfowl, and viruses of class II, the majority of which are virulently sourced from poultry, was compared, differences in codon usage within the fusion gene and in the full genome of the different classes were found. This suggests that NDV has the capacity to utilize this mechanism for adaptation.

In order to estimate the time to the most recent common ancestor of class I and class II viruses and between best-represented genotypes within class II, a Bayesian Inference approach was implemented using the BEAST package [63] by Taylor [62]. As a result, it was shown that class I and class II APMV-1 have evolved from ancestors that existed between 1772 and 1782, and the observed differences in codon usage probably reflected a period of approximately 234–244 years of evolution. For that period of evolutionary time a low Pearson’s r coefficient of 0.35 was observed when comparing the relative synonymous codon usage values of class II versus class I sequences. This lack of correlation suggested the existence of adaptive codon usage evolution in NDVs.

In contrast with previous results, a Pearson’s r correlation coefficient of 0.8 was observed when analyzing the relative synonymous codon usage values among different viruses within class II. Class II viral fusion gene sequences higher positive correlation suggested that fewer codon usage changes have occurred among recently evolved viruses. Among virulent viruses normally expected to be maintained in chickens (genotype VII) versus mesogenic viruses that are normally maintained in pigeons (genotype VI), there was an evolutionary estimate time to the most recent common ancestor of approximately 69–73 years; thus, in agreement with the idea of a slow process of codon usage adaptation, the observed differences in codon usage these among class II viruses were not as significant as the differences observed between classes. The analysis concluded that codon usage adaptation is likely to be active, but it is a slow process that is not easily measured in short evolutionary periods.

**Figure 2:**
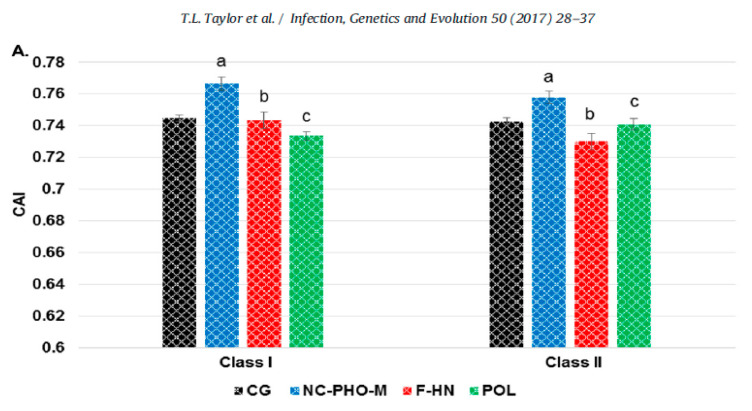
Codon usage adaptation in genomes of class I and II viruses. Excerpts from Figure 2 in Taylor, T.L.; Dimitrov, K.M.; Afonso, C.L. Genome-wide analysis reveals class and gene-specific codon usage adaptation in avian paramyxoviruses 1. [62]. Avian paramyxoviruses 1 display differences in codon usage between the three transcriptional genomic regions. A) The average codon adaptation index (CAI) value for the complete genome and each genomic region (NC-PHO-M, F-HN, and POL) for class I (*n* = 29) and class II (*n* = 259) viruses was determined using *Gallus gallus* as a reference genome. The complete genome result is in black, NC-PHO-M is in blue, F-HN is in red, and POL is in green. Standard error bars are shown, and statistically significant differences between the same regions between classes are shown (Pb 0.01) with corresponding letters.

Another finding of the study was that the codon usage bias for the region encoding the F and HN genes were distinct from the codon usage bias of each one of two other genomic regions (Figure 2). The average codon adaptation index (CAI value) of the complete genome and of each of three genomic regions that code functionally distinct genes (NC-*P-*M, F-HN, and P) for class I (*n* = 29) and class II (*n* = 259) viruses, was determined using *Gallus gallus* as the reference genome. The class II F and HN are surface proteins that have already been shown to be affected by positive selection and by antibody-selective pressures and encode genes with clear functional differences with genes encoded in other genomic regions (Figure 2) [48,49]. It is possible, therefore, to expect differences in the evolutionary rates at those two genes in comparison to the evolution at other more internal genes such as the M, NC or P. This observation is relevant because the evolution of the F and HN proteins in the class II viruses is likely to be influenced by intensive vaccination in poultry. As the F gene encodes for a surface protein that contains the sites for neutralization, it is possible that the differences seen in codon usage between the surface proteins and other genomic regions may be partially attributed to selection caused by immune pressure.

### 2.3. Modulation of Virulence during Host Adaptation

**Figure 3:**
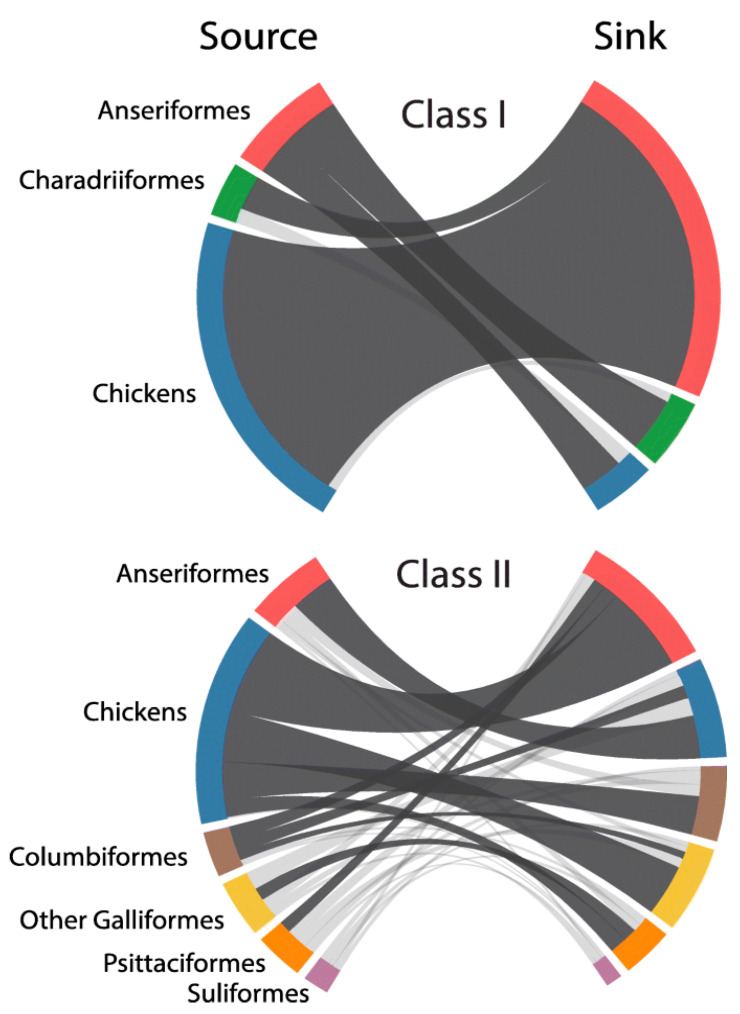
Inter-host transmission of Newcastle disease viruses (NDV). Excerpts from Figure 2 in Hicks, J.T.; Dimitrov, K.M.; Afonso, C.L.; Ramey, A.M.; Bahl, J. Global phylodynamic analysis of avian paramyxovirus-1 provides evidence of inter-host transmission and intercontinental spatial diffusion [64] Class I and class II chord diagrams representing the fully resolved transition matrix between host orders. Chord width between source and sink host state is proportional to the median transition rate per year. Dark gray chords are statistically supported (BF > 3.0). Colors correspond to host order: Anseriformes—red, Charadriiformes—green, domestic chickens—blue, Columbiformes—brown, other Galliformes—yellow, Psittaciformes—orange, Suliformes—purple.

The hypothesis that NDV viruses modulated virulence during host adaptation requires evidence of the occurrence of different types of evolutionary events such as (1) inter-host transmission; (2) origin of recent virulent viruses from older virulent viruses; (3) genetic changes consistent with adaptation to maintenance in different hosts; and (4) demonstration of changes in virulence-associated with these genetic changes. Modern techniques of phylogenetic analysis provide evidence for events 1, 2 and 3, and comparison among the pathogenicity and virulence of viruses adapted to different hosts provides evidence for type 4 events. Although many reports support the capacity of NDV to infect multiple hosts, a recent global phylodynamic analysis of avian paramyxovirus-1 done by J. T. Hicks using the complete fusion protein coding sequence has provided sufficient statistical evidence supporting NDVs capacity for inter-host transmission [64]. Hicks’ study (Figure 3) revealed that, in general, the ancestral history of class II viruses was structured by the host, with host types clustering together within genotypes. For instance, genotypes XII, XIII, XIV, XVII and XVIII were predominantly detected in domestic chickens with evidence for few transitions to other hosts, including Anseriformes, Psittaciformes, Columbiformes and other Galliformes birds. In contrast, genotypes I and VI were mostly detected in non-chicken hosts, such as Anseriformes and Columbiformes, respectively.

This nucleotide-based analysis demonstrated the capacity for NDV to transmit among divergent host taxa over time (Figure 3). The highest (most strongly supported) transition rate for viruses of class I and II existed from domestic chickens to Anseriformes, and among class II viruses for domestic chickens acting as a source for Columbiformes, other Galliformes, and Psittaciformes. Columbiformes was also highly supported sources of viruses to Anseriformes and domestic chickens. Notwithstanding the evidence of the existence of different transmission rates of NDV among different hosts species; some restrictions appeared to exist as virulent NDVs were not repeatedly isolated in migratory waterfowl populations (including birds of the Anatidae family ducks, swans, geese or other species with flattened beaks and fully webbed toe). Despite the abundant sampling (normally done for AIV surveillance) and of the existence of large populations of waterfowl and shorebirds in the wild, there is no strong evidence of virulent viruses being maintained in these types of birds. With only one isolated case of increased virulence for a class I virus ever reported [65], the data suggest that there may be genetic or ecological restrictions preventing the development and maintenance of highly virulent viruses in the class I viruses adapted to shorebird and waterfowl.

Demonstration of the origin of recent virulent viruses from older virulent viruses (2) and evidence of genetic changes consistent with adaptation to maintenance in different hosts (3) can be obtained by phylogenetic analysis of large databases. The genetic diversity of current NDV class II virulent genotype viruses was recently studied by an international consortium [22]. For this study, a total of 1956 complete F gene sequences were used; this included viruses of class I (*n* = 284) and class II (*n* = 1672). Detailed tables of all available sequences, including genotypes, host, date and place of isolation, are available in Supplemental Tables S1 and S2 of Dimitrov 2019 manuscript [22]. Conclusions were based on neighbor-joining, maximum-likelihood, and Bayesian method-based trees represented in their Supplementary Figure S4 for class I and Figure S5 for class II viruses (Supplementary Figures S1 and S2 of this manuscript are excerpts from Dimitrov’s Figure S5) [22]. Phylogenetic analysis of the fusion protein-coding region and complete genomes reaffirmed the evolution of NDVs into two large classes (class I and II). The viruses of class I were predominantly reported in wild waterfowl and shorebirds, and viruses of class II were preferentially isolated from chickens but also present in some wild bird species. Class I viruses werer lentogenic and more conserved, clustering into just one genotype, and despite the diversity in hosts, less than 10% nucleotide differences were observed among isolates. Class II viruses were between 41.0 to 46.3% genetically divergent from the class I viruses, contained the majority of virulent viruses, and had larger genetic diversity with 20 distinct genotypes and nucleotide distances among genotypes ranging from 7.8 to 28.9%.

**Figure 4:**
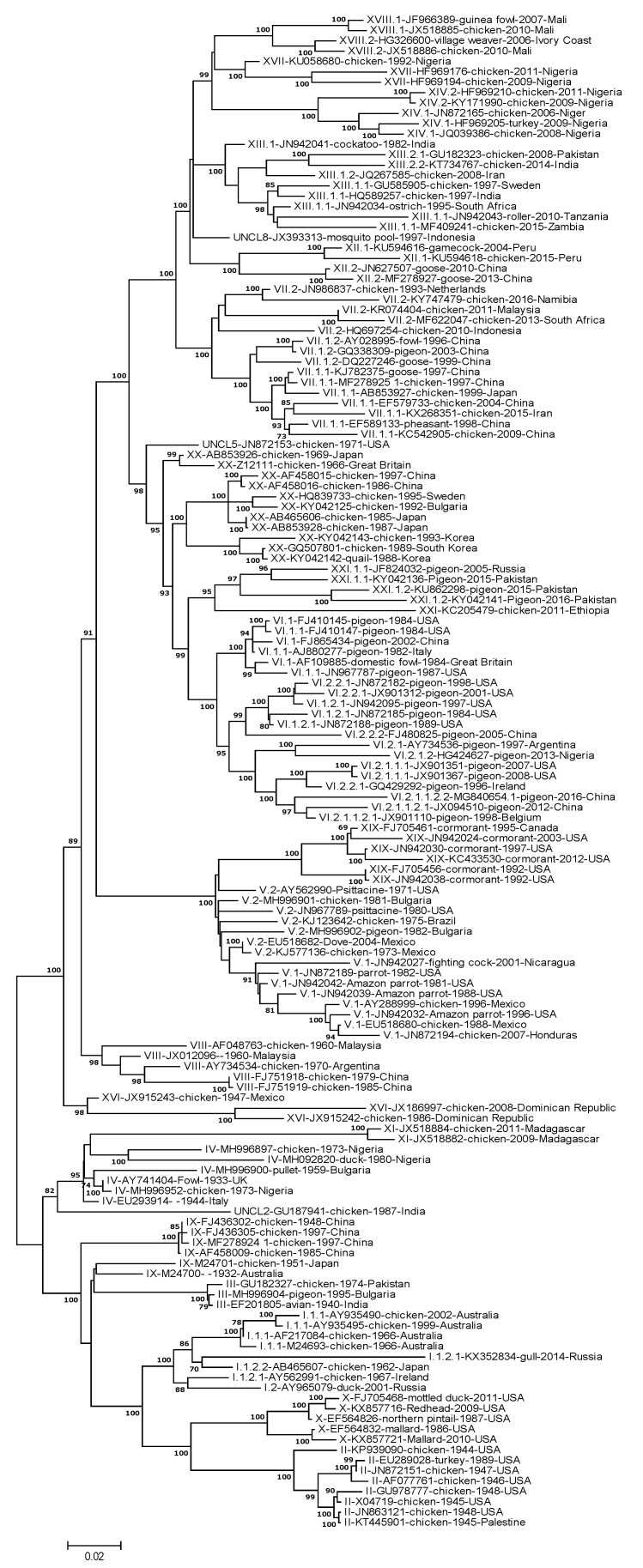
Molecular phylogenetic analysis of NDVs representing historical and current genotypes by the maximum likelihood method. The evolutionary history was inferred by using the maximum-likelihood method based on the general time reversible model. The tree with the highest log likelihood (−32,597.46) is shown. Virus description in the tree includes genotype classification, accession number, host, year of isolation and country as described by Dimitrov [22]. The percentage of trees in which the associated taxa clustered together is shown next to the branches. Initial tree(s) for the heuristic search were obtained automatically by applying neighbor-join and BioNJ algorithms to a matrix of pairwise distances estimated using the maximum composite likelihood (MCL) approach and then selecting the topology with superior log likelihood value. The tree is drawn to scale, with branch lengths measured in the number of substitutions per site. The analysis involved 146 nucleotide sequences. Codon positions included were 1st+2nd+3rd+noncoding. All positions containing gaps and missing data were eliminated. There were a total of 1653 positions in the final dataset. Evolutionary analyses were conducted in MEGA7 [69].

Excerpts in Supplementary Figure S1 here are taken from Dimitrov’s full phylogenetic trees based on the complete fusion gene sequences of isolates representing NDVs of class II (*n* = 1672). In the phylogenetic trees produced by Dimitrov’s study, the origin of current virulent viruses can be traced back to the oldest characterized virulent genotypes circulating during the 1930s and 1940s. These include the genotype III virus identified as Australia 1932 and chicken Mukteswar 1940 as the oldest ancestors in Asia. The genotype IV oldest viruses in Europe were the Herts isolated in chickens in the United Kingdom in 1933 and the Italien isolated in Italy in 1944 (Figure 4 and Supplementary Figure S1). In the Americas, the oldest vNDVs were members of genotype XVI and II; an example of this is the genotype XVI virus 1947 Queretaro from Mexico. In addition, the 1944 chicken California virulent virus is the oldest relative of multiple virulent viruses of genotype II isolated throughout the US. In China, the older isolates are viruses from genotype IX from 1948, forming a genotype that appears to be extinct in the wild. The phylogenetic analysis revealed that older origin genotypes, some of which are no longer circulating (e.g., genotypes II, III, IV, IX), likely may have provided indirect ancestry to present-day viruses giving rise to other genotypes such as genotype VIII.

Key for this analysis is the well supported (by bootstrap analysis of 97 in the maximum-likelihood trees, and by posteriors of 1 in the Bayesian trees) ancestral relationship of with genotype VIII chicken viruses (1960–1970) for viruses representing nearly all of the most recent cormorant, pigeons, and vvNDV genotypes of chickens (In Figure 4 and Supplementary Figure S1 with a bootstrap of 89). The ancestral genotype VIII viruses were apparently worldwide distributed and included isolates from 1960 to 1970s such as 1960 AF2240 Malaysian, China_QH4_1985, and Argentinian Trenque Lauquen 1970–1971 and characterized as vvNDV. With the exception of genotypes I, II, III, IV, IX, X and XVI, all the viruses representing recent genotypes shared a common ancestor with viruses of genotype VIII.

Genotype XIII in East Asia and Africa appear to share a common ancestor with the virus India 1982. Genotype VII, which is highly distributed in chickens across Asia and detected in Africa Europe causing occasional mortality in waterfowl, had older reported common ancestors with Chicken Netherland 1993 and in chicken Taiwan 1995; genotype XII had an ancestor related to Mosquito Pool Indonesia 1997; in Africa, the related genotypes XIV, XVII and XVIII seem to have Nigeria Kudu 1992 as the older ancestor; in Madagascar viruses of genotype IX have distantly related ancestors related to genotype IV circulating during the 1940s. Overall, phylogenetic analysis, in addition to supporting the origin of most current virulent lineages from older virulent poultry viruses, also supports the origin of mNDV of pigeons and vnNDV of cormorants from old virulent viruses of poultry (Figure 4 and Supplementary Figure S1).

Adaptation of chicken virulent NDV to pigeons suggest modulation of virulence. Virulent NDVs preferentially associated with pigeons have been commonly referred to as pigeon paramyxoviruses or PPMV-1 (originally including only genotype VIb, but more recently including other lineages of pigeon origin). Pigeon origin viruses have been a part of the panzootic in Columbiform birds that emerged more than four decades ago, with these viruses still circulating worldwide [35,36,37,39,40,66]. Viruses of pigeons are capable of infecting chickens and domestic or wild pigeons. Highly related viruses of this group were recently re-classified as members of genotypes VI, XX and XXI. Genotypes VI and XXI genotypes are preferentially represented by pigeon isolates, but genotype XX, previously referred to as VIc, is an older genotype that was reported to circulate in east Asia in chickens from 1980 to the 1990s. The oldest isolate in the group is the Chicken Japan 1985 virus. The phylogenetic reconstructions by Dimitrov et al. demonstrate that older isolations of genotype VI viruses, such as domestic Pigeon Italy 1982, Great Britain domestic Fowl 1984, and USA pigeon 1987, were highly related to chicken ancestral isolates from the 60s and 70s such as the Great Britain Warwick in 1966, the Japan Osaka, 1969 and the USA as California Fontana 1971 isolates (Figure 4 and Supplementary Figure S2A). Viruses from these genotypes are recovered predominantly from pigeons but occasionally are found in kestrels, falcons, cockatoos, budgerigars, pheasants, etc., in numerous locations throughout the world. The abundance of older chicken isolates in genotype XX and the closer relationship of this lineage with other older lineages from chicken suggests that the pigeon lineages may have originated from a chicken adapted lineage. A similar observation was made by Meulemans et al. in 1987, who showed that polyclonal and monoclonal antibodies directed against the HN and F proteins of the chicken velogenic Italien NDV reacted with 21 pigeon isolates, thus showing a close phylogenetic relationship between old chicken viruses and pigeon viruses [67,68].

vnNDV and mNDV that infect cormorants and other species of the genus Phalacrocorax) in North America represent the second group of wild bird viruses that may have originated from a common ancestral virus from chickens and adapted by reducing virulence (Figure 4, Supplementary Table S1 and Figure S2B). Viruses of cormorants are found almost exclusively on wild birds sampled in the United States and Canada (with the exception being detection in a North Dakota free-range turkey farm in 1992) and are very closely related to other viruses of genotype V established in chickens and responsible for the second ND panzootic. Phylogenetic analysis reveals that cormorant viruses likely emerged in Central and/or South America in the 1970s (Figure 4, Supplementary Table S1 and Figure S2B). Cormorant viruses are a present-day lineage that causes mortality in wild cormorants and has been better documented in young, hatch-year, naïve cormorants [32,34,70,71]. They belong to the recently renamed genotype XIX (previously Va), sharing a common ancestor with other vvNDV of genotype V such as the Psittacine Largo Florida 1971, Chicken Brazil 1975 and Parrot Argentina 1975.

The factors important for the apparent virulence adaptation seen in pigeons and cormorants’ viruses are unknown. Although speculative, one would expect that host species that form long-term associations, or high-density colonies in which proximity is granted would favor certain replicative features of virulent viruses that have previously adapted to replication in poultry, such as a capacity to transmit only in close proximity with long term contact periods. Mass nesting is common in pigeons, with dozens of birds sharing buildings in which nests and droppings tend to stay clustered and remain dry when out of the weather. Cormorants are generally gregarious, nesting in colonies, gathering in flocks and often hunting together in groups, which sometimes numbers up to 4000 birds. Other species that congregate in larger groups, such as parrots, have also been reported to carry virulent viruses. The isolation of highly virulent viruses of genotype V from psittacine in the past has suggested a possible role as wild bird reservoirs for highly virulent viruses. However, the persistence of those psittacine isolations over time has not been demonstrated. It remains to be seen if herons, egrets, flamingos, albatrosses, penguins, swallows, oropendolas, and other colonial birds may also be receptive to the maintenance of virulent viruses currently circulating in chickens. In infections of wild avian populations (such as those of pigeons and cormorants) with lower or non-existent levels of immunity in non-infected birds, it may not be efficient to maintain highly lethal viruses, and further evolution toward a lower level of virulence may be a more effective viral strategy for achieving fitness. However, those theories need to be further investigated.

Although an initial inference of changes in virulence during evolution can be indirectly achieved by analyzing the prevailing ICPI of viruses representing different genotypes, the ICPI test often lacks the resolution to distinguish changes in virulence affecting adult birds. Consequently, changes in virulence as an evolutionary host adaptation mechanism are best studied doing pathogenicity studies in adult birds. Table 1 is a selected compilation of ICPI values from the literature on representative viruses of different genotypes. In Table 1, differences in ICPI according to data and host of origin are presented, and the differences were seen between older recent chicken viruses and wild bird adapted viruses suggest that virulence is a fluctuating evolutive characteristic of NDV. The majority of recent chicken viruses and a few older viruses have ICPI values > than 1.5, while the most recent viruses of cormorants and pigeons are mesogenic with ICPI values < or close to 1.5. ICPI of viruses of cormorant range between 1.3 to 1.6 and from wild pigeons from 0.9 to 1.3. Representative viruses of genotype V, VII, XII and XVI that are highly adapted to chickens and shown to be vvNDV have ICPI values greater than 1.6.

**Figure 5:**
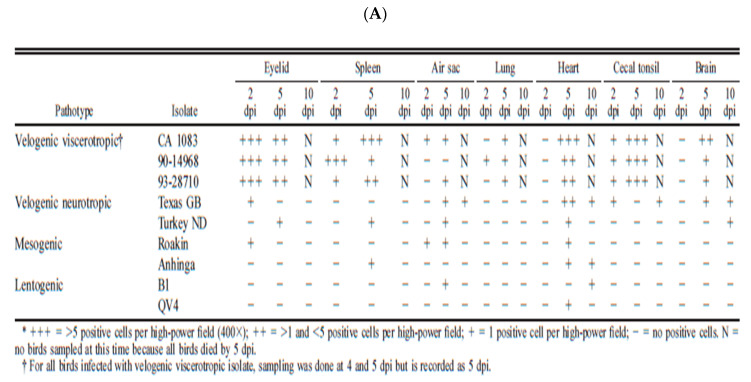

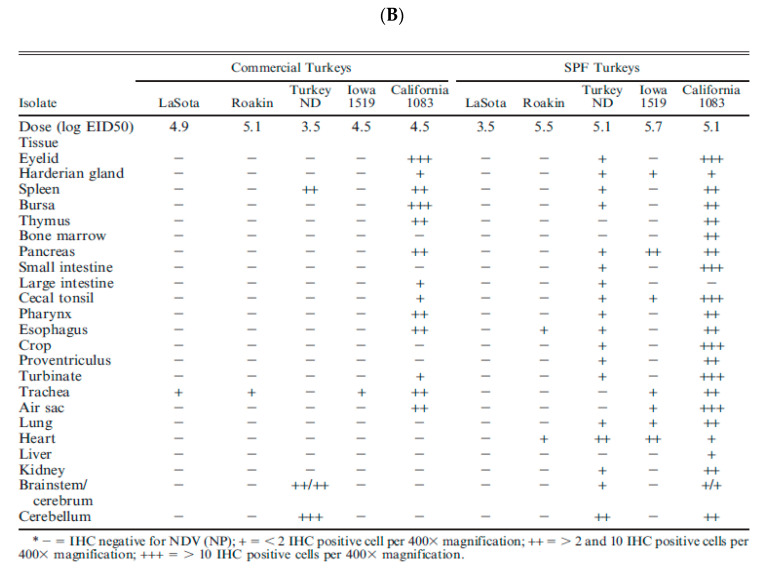
Pathogenesis comparison between vvNDV and vnNDV. (**A**) Excerpts from Brown, C.; King, D.J.; Seal, B.S. Pathogenesis of Newcastle Disease in Chickens Experimentally Infected with Viruses of Different Virulence. [16]. (**B**) Excerpts from Piacenti, A.M.; King, D.J.; Seal, B.S.; Zhang, J.; Brown, C.C. Pathogenesis of Newcastle disease in commercial and specific pathogen-free turkeys experimentally infected with isolates of different virulence [73].

Despite the ICPI values shown in Table 1, the differences between neurotropic and viscerotropic viruses of different origins are best determined by performing pathogenesis studies in adult naïve pathogen-free birds. Many studies have been conducted to demonstrate the damage of vvNDV in naïve chickens. However, few have been done in a comparative manner to evaluate the differences in virulence between vnNDV and vvNDV. Figure 5A,B are excerpts from publications from Brown and Piacenti [16,72,73].

Pathogenicity studies in adult-specific pathogen-free chickens were used to demonstrate that viruses classified as vvNDV have increased virulence in comparison with vnNDV viruses. The studies by Brown and Piacenti demonstrate that the viruses from both pathotypes can target cells of the brain and cause mortality. However, a key difference between vvNDV and vnNDV was more rapid mortality in chickens infected with vvNDV (4–6 days) as one of the factors that prevent the development of neurogenic damage (which normally occurs later at days 8–10). The increased virulence of vvNDV in CA 1083, 90-14698, and 93-28710 isolates was characterized by acute systemic illness, extensive necrosis of lymphoid areas in the spleen and intestine, neurological damage with rapid mortality (Figure 5A,B). Instead, the vnNDV (Texas GB, and turkey ND)-infected chickens had lower damage and survived longer, making it possible for the damage of the central nervous system to take place, despite the reduced amounts of viral nucleic acid detected initially in neural tissue. These studies suggested that vnNDV were indeed less virulent than more recent vvNDV isolates, and they argue for a prevalence of viruses of increased virulence in recent years.

**Figure 6:**
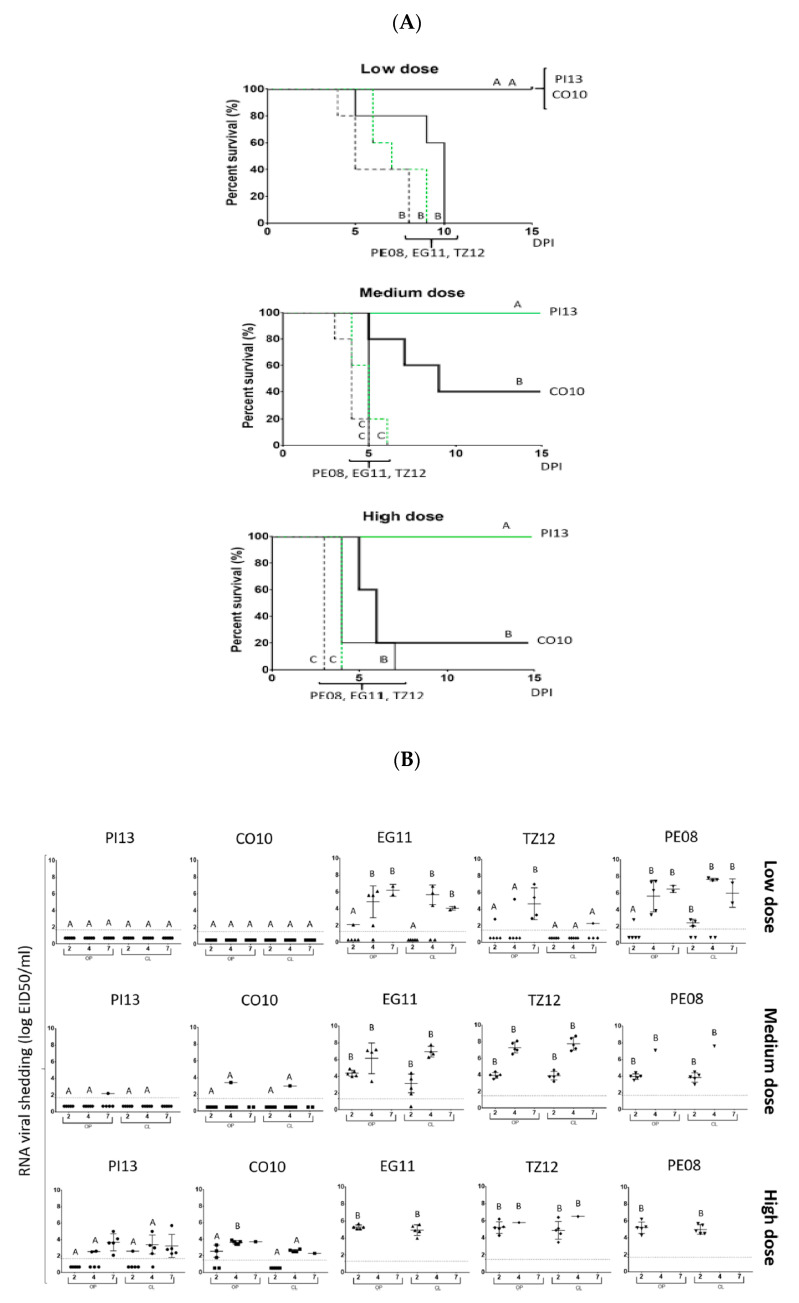
Comparison of the capacity for virulence, replication and shedding between chicken and wild bird isolates. Excerpts from Figures 1 and 3 in Ferreira, H.L.; Taylor, T.L.; Dimitrov, K.M.; Sabra, M.; Afonso, C.L.; Suarez, D.L. Virulent Newcastle disease viruses from the chicken origin are more pathogenic and transmissible to chickens than viruses normally maintained in wild birds [55]. (**A**) Survival curves of directly inoculated birds. Chickens were separated into 3 groups for each virus and inoculated with a low, medium, and high dose of the five NDV strains (PE08, EG11, TZ12, CO10, PI13). Mortality in each experimental group was followed daily over 14 days. No common letters indicate significant differences (*p* < 0.5). (**B**) Virus shedding is directly inoculated birds after inoculation with chicken- and wild bird-origin NDV. NDV titers were estimated in both oropharyngeal (OP) or cloacal swab (CL) swabs of directly inoculated birds with three different doses of NDV strains at 2, 4, and 7 DPI. The detection limit of the different RRT-PCR assays targeting the NDV strains varied between 1.5 and 1.7 log10EID50/mL and are shown as dotted lines on the Y-axis. Mean and standard deviation of the mean for positive swabs at each time point are shown as bars. No common letters (A or B) differ significantly (*p* < 0.05) when comparing oropharyngeal or cloacal swab samples from the different viruses with the same infectious dose and same sampling point. Non-detected swabs were added below the limit of detection for each virus.

Additional evidence for changes in virulence during adaptation to different hosts was demonstrated by Ferreira and others who compared virulence and transmission of NDV of different origins [55]. In these studies, recent chicken origin viruses were more virulent and transmitted better than wild bird viruses from pigeons and cormorants. Studies were performed only on chickens due to the difficulties in obtaining specific pathogen-free (SPF) wild birds. The authors conducted challenge experiments in SPF naïve chickens and demonstrated a different capacity of viruses of different origin to replicate and re-infect. Phylogenetic analysis of the viruses studied in this experiment demonstrated that the recently isolated vvNDV viruses clustered into five different class II genotypes. An Egyptian vvNDV clustered with poultry viruses isolated between 2012 and 2017 belonging to genotype VII, a Peruvian vvNDV from 2008 grouped with other poultry viruses isolated from Peru in 2011 and a vvNDV from Colombia in 2009 and is classified as XII. A Tanzania virus clustered with isolates from several African countries isolated during 2008–2015 and belongs to genotype XIII. The wild bird adapted viruses include a cormorant virus that clustered with cormorant isolates from the USA from 2010 to 2012 and is classified as XIX. The pigeon adapted virus clustered with samples from pigeons and doves in Pennsylvania and Massachusetts from 2012 and 2014 and belongs to genotype VI. In Ferreira’s work, chicken-origin vvNDV killed chicken faster, secreted more viruses, were capable of more efficiently transmit to direct contact controls and had a much lower CID50/mL than viruses of wild bird origin from pigeons and cormorants. Figure 6A,B. Figure 6 presents excerpts from Figures 1 and 3 in the Ferreira, H.L 2019 article [55].

Mortality rates, clinical signs, and gross lesions observed in birds infected with the chicken-origin vvNDV were similar to those reported previously for other vvNDV [74,75]. Chicken-origin NDVs sustained high levels of replication in chickens, usually with increased virus titers through 4 DPI, and infected chickens showed severe depression, and all died or were euthanized because of severe clinical signs by day 5 post-inoculation. In these birds, histologic lesions were widespread, and the virus was detected in multiple organs typically shortly before death. In the present study, the low CID50/mL, efficient transmission, and fast mortality for all three chicken origin viruses indicated that these viruses were highly adapted to chickens with major differences in the CID50/mL observed between the chicken-origin and wild bird-origin NDV and the need for a higher dose for wild bird NDV to infect chickens.

### 2.4. Role of Host Associated Evolutionary Factors

The role of host-associated evolutionary pressures is a neglected area of research that needs to be further investigated. The need to increase the frequency of poultry vaccination against NDV in recent years in endemic countries to control the constant presence of vvNDV suggests that evolution toward increased virulence in chickens may be a byproduct of current poultry production systems [76]. Immunized animals such as vaccinated poultry are expected to be capable of tolerating infections with more virulent viruses and of requiring higher infectious doses. Human activities that create large reservoirs of closely confined birds, transcontinental mobility through trade or pigeon racing, or increased survival without eliminating viral replication and shedding during vaccination are all likely to create new opportunities for NDV evolution. Reported spillover of vaccine-derived Newcastle disease viruses into different species of wild birds across four continents from 1997 through 2014 [77] and of chicken-virulent viruses (Mexico, Pakistan, Nigeria, Israel, China, etc.) from chickens into wild birds [78,79,80,81,82] support a human role on NDV evolution. Viruses maintained in wild bird species may, in contrast, require the development of other adaptations such as resistance to UV or desiccation or the ability to survive in an aquatic environment that may not necessarily promote virulence. On the other hand, viruses maintained in highly vaccinated poultry would be expected to be environmentally more fragile (as bird-to-bird proximity in farms reduces the need of the virus to survive in the environment), to be more host restricted (as there is no need to jump into intermediate hosts); more virulent, and more resilient against immune responses. Thus, the genetics of the host, farm management, vaccination, environment, mode of dissemination, social habits, and human-created agroecological environments may all contribute with selective pressures in a multidimensional and complex evolutionary process that affects directly or indirectly virulence.

## 3. Conclusions

The role of virulence on host adaptation (or vice versa) during more than ninety years of NDV evolution was analyzed. The data suggests that recent virulent viruses from wild birds originated from older chicken virulent viruses and indicates that the maintenance of virulent NDV in chickens or in wild birds may have led to the modulation of virulence in opposite directions. In evolutionary terms, the evolution of virulence in different directions is expected to occur [46,83,84]. Recognizing that chicken vNDV i capable of becoming established in wild birds and of modulating virulence during adaptation to new hosts is a first step toward developing effective control strategies.

## Figures and Tables

**Table 1:** Intracerebral pathogenicity index (ICPI) of selected viruses sorted by genotypes.

Year	ICPI	Genotype	Host	Country	Isolate Name	GB Acc.
2001	0.2	I.1.2.1	Redknot	USA	A_101_1383	EF564816
1948	1.8	II	Chicken	USA	TX_GB	GU978777
1948	1.5	II	Chicken	USA	New_Jersey_Roakin	JN863121
1945	1.45	II	Chicken	USA	Beaudette_C	X04719
1946	0.4	II	Chicken	USA	Lasota	AF077761
1947	0.2	II	Chicken	USA	Hitchner_B1	JN872151
1989	0.03	II	Turkey	USA	VG_GA	EU289028
1971	1.8	V	Chicken	USA	Ca_2098	JQ247691
2002	1.79	XIX	Chicken	USA	CA_212676	EF520718
2000	1.64	XIX	Chicken	Mexico	Chicken-Torreon_453	EU518677
2000	1.61	XIX	Chicken	Honduras	Chicken-15	AY288993
2008	1.66	V.2.1	Turkey	Belize	4438_4	JN942045
2005	1.89	V.2	Chicken	Mexico	NDV_P05	HM117720
2006	1.91	V.2	Chicken	Mexico	Estado_de_Mexico_466	EU518684
2004	1.94	V.2	Chicken	Mexico	Distrito_Federal_462	EU518682
2005	1.99	V.2	Chicken	Mexico	Estado_de_Mexico_465	EU518683
1973	1.93	V.2	Dove	Mexico	Chimalhuacan	KJ577136
2009	1.65	V.2_	Macaw	Mexico	Chiapas_672_ZM12	KC808510
1971	1.76	V	Psittacine	USA	Largo	AY562990
2005	0.98	VI.1.2.1.1.1	Dove	USA Texas	TX4156	EU477192
2000	1.13	VI.1.2.1.1.1	EC_Dove	USA RI	RI166	EU477189
2004	1.15	VI.1.2.1.1.1	EC_Dove	USA Texas	TX3503	EU477190
2013	1.15	VI.1.2.1.1.1	EC_Dove	USA	Allegheny_PA_ND0007199	KP780874
2013	1.35	VI.1.2.1.1.1	EC_Dove	USA	Allegheny_PA_ND0007186	KP780872
2005	1.26	VI.1.2.1.1.1	Pigeon	USA Texas	TX3988	EU477191
2004	1.3	VI.1.2.1.1.1	Pigeon	USA TX	TX3988	KU059752
2013	0.66	VI.1.2.1.1.1	Rock_Pigeon	USA	Ingham_MI_ND0003553	KP780875
2006	0.73	VI.1.2.1.1.1	Rock_Pigeon	USA	TX6295	EU477195
2004	0.79	VI.1.2.1.1.1	Rock_Pigeon	USA Texas	TX_B_2580	EU477188
2013	0.88	VI.1.2.1.1.1	Rock_Pigeon	USA	Ingham_MI_ND0003558	KP780876
2013	1.13	VI.1.2.1.1.1	Rock_Pigeon	USA	Allegheny_PA_ND0007190	KP780871
2013	1.15	VI.1.2.1.1.1	Rock_Pigeon	USA	MD_ND0002270	KP780870
2013	1.2	VI.1.2.1.1.1	Rock_Pigeon	USA	Allegheny_PA_ND0007187	KP780873
1971	1.76	VI	Chicken	USA	California_1083_Fontana_	JN872153
2009	1.86	VII.1.1	Chicken	Venezuela	VEN-611	JQ319052
1987	0.1	X.1	Northern_Pintail	USA	US_OH_87_486	EF564826
1999	0	X.2	mallard	USA	US_MN_99_376	FJ705466
1986	0	X.2	mallard	USA	US_OH_86_233	EF564832
2011	1.78	XII.1	Chicken	Peru	Peacock	KR732614
2008	1.8	XII.1	Peacock	Peru	1918_03	JN800306
1995	1.39	XIX	Cormorant	Canada	95DC2345	FJ705461
1995	1.39	XIX	Cormorant	Canada	95DC02150	FJ705460
2008	1.39	XIX	Cormorant	USA	503	GU332662
1998	1.41	XIX	Cormorant	Canada	98CNN3_V1125	FJ705459
2010	1.43	XIX	Cormorant	USA	ME_659	JN255782
2005	1.45	XIX	Cormorant	USA	NV_19529_04	FJ705463
2010	1.45	XIX	Cormorant	USA	MA_651	JN255775
2010	1.51	XIX	Cormorant	USA	ME_658_	JN255781
2008	1.53	XIX	Cormorant	USA	496	GU332655
2010	1.53	XIX	Cormorant	USA	MN_648	JN255784
1997	1.53	XIX	Cormorant	USA CA	CA_D9704285	FJ705458
2010	1.54	XIX	Cormorant	USA	HN_652	JN255785
2010	1.59	XIX	Cormorant	USA	656	JN255779
1997	1.6	XIX	Cormorant	USA	CA_97_23071	FJ705457
1992	1.38	XIX	Gull	USA	MN_92_40140	FJ705456
2010	1.39	XIX	Gull	USA	655	JN255778
2008	1.88	XVI	Chicken	Dominican Republic	499_31	JX119193

## Supplementary Materials

The following are available online at https://www.mdpi.com/1999-4915/13/1/110/s1, Figure S1: Phylogenetic relationships among viruses of older genotypes (II, III, IV, IX). Figure S2: Relationship between viruses of pigeons and cormorant with chicken viruses, Table S1: Information on genotype, accession numbers, hosts, country, isolate name, year of isolation and sequences corresponding to Figure 4.
