# Supplementary material for: Virulence during Newcastle Disease Viruses Cross Species Adaptation
Source: Viruses. 2021 Jan 15;13(1):110. doi: 10.3390/v13010110 (PMC7830468; doi:10.3390/v13010110)
Supplement: Supplementary file 1 [file viruses-13-00110-s001.zip › viruses-1042536-supplementary/supplementary-revised/Supplementary Figure 1.docx]

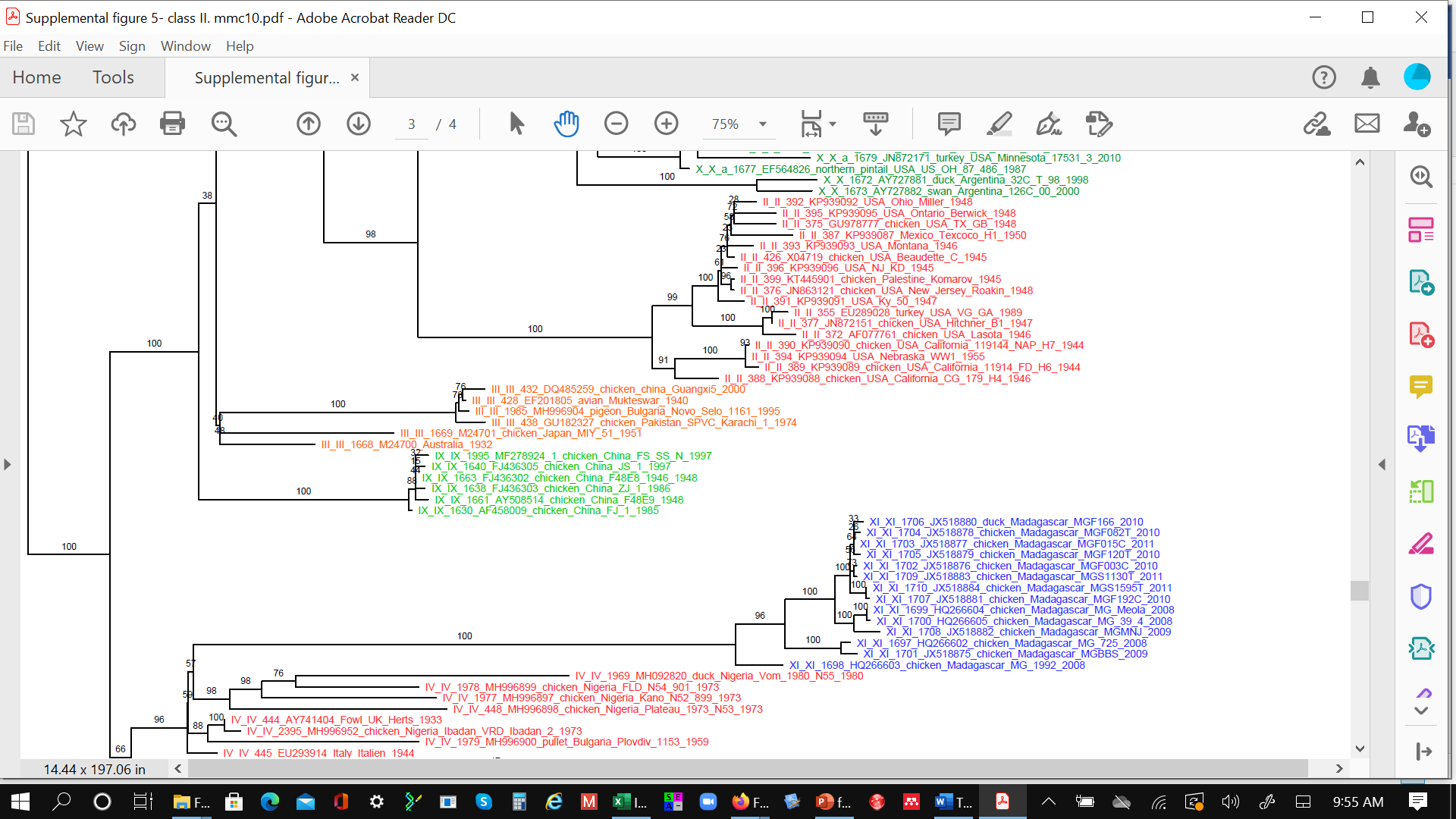


**Supplementary Figure 1. Phylogenetic relationships among viruses of older genotypes (II, III, IV, IX).** Excerpts from [22]. Updated unified phylogenetic classification system and revised nomenclature for Newcastle disease virus. Infection, genetics and evolution 2019, 103917 in Supplemental Fig. S5B Class II Maximum Likelihood tree. Different colors indicated different genotypes.
