# Supplementary material for: Virulence during Newcastle Disease Viruses Cross Species Adaptation
Source: Viruses. 2021 Jan 15;13(1):110. doi: 10.3390/v13010110 (PMC7830468; doi:10.3390/v13010110)
Supplement: Supplementary file 1 [file viruses-13-00110-s001.zip › viruses-1042536-supplementary/supplementary-revised/Supplementary Figure 2.docx]

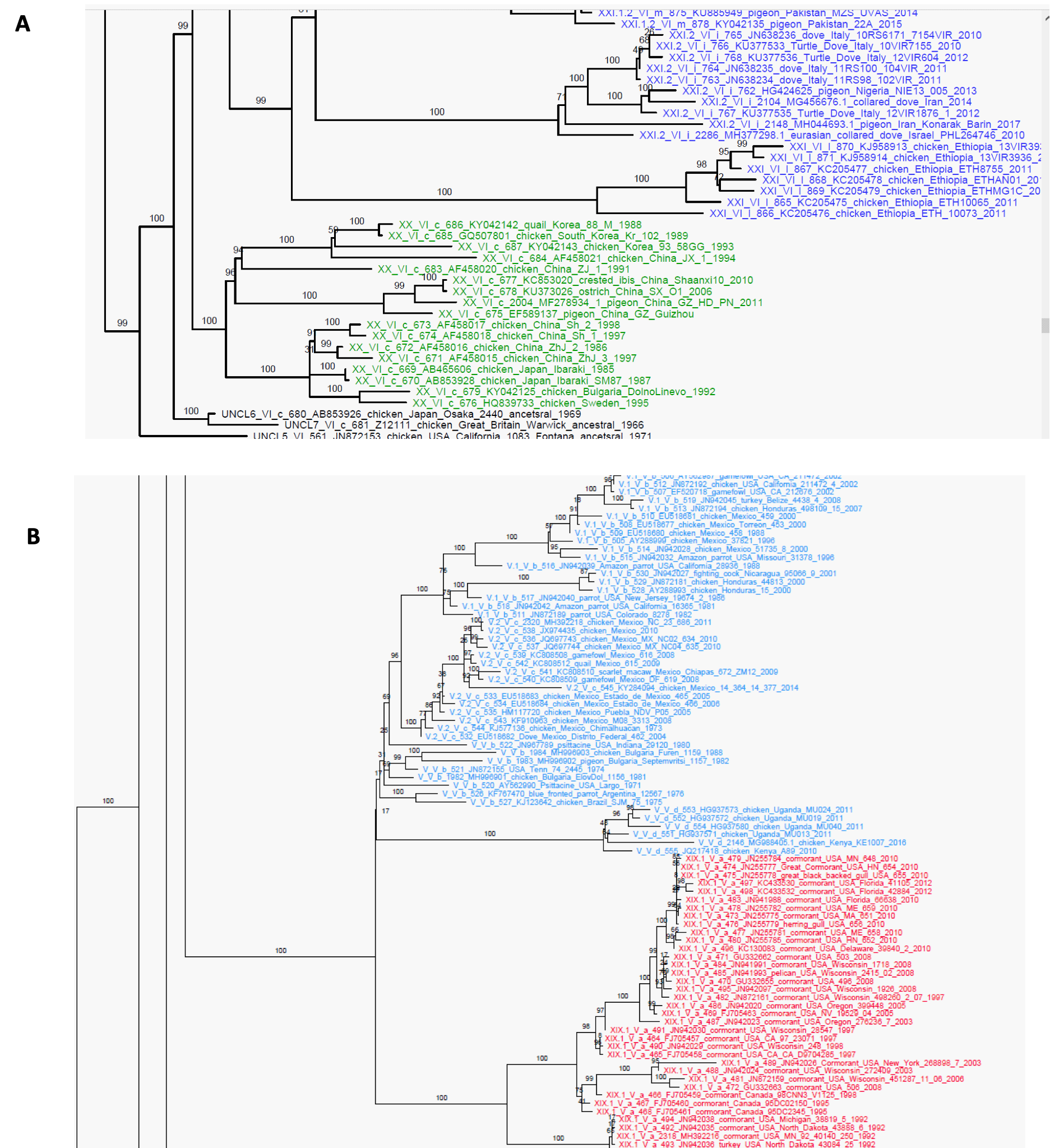


**Supplementary Figure 2. Relationship between viruses of pigeons and cormorant with chicken viruses.** Excerpts from [22]. Updated unified phylogenetic classification system and revised nomenclature for Newcastle disease virus. Infection, genetics and evolution 2019, 103917, in Supplemental Fig. S5B Class II Maximum Likelihood tree. **A.** Relationship among pigeon viruses of genotype XIX, ancestral virulent chicken viruses of genotype XX, and virulent viruses circulating in the 60s. **B.** Relationship among cormorant virus of genotype XIX and virulent viruses of chickens from genotype V circulating in the 70s and 80s.
